# Genome-Wide Analysis of DNA Methylation in Human Amnion

**DOI:** 10.1155/2013/678156

**Published:** 2013-02-19

**Authors:** Jinsil Kim, Mitchell M. Pitlick, Paul J. Christine, Amanda R. Schaefer, Cesar Saleme, Belén Comas, Viviana Cosentino, Enrique Gadow, Jeffrey C. Murray

**Affiliations:** ^1^Department of Anatomy and Cell Biology, University of Iowa, 500 Newton Road, 2182 ML, Iowa City, IA 52242, USA; ^2^Department of Pediatrics, University of Iowa, 500 Newton Road, 2182 ML, Iowa City, IA 52242, USA; ^3^Departamento de Neonatología, Instituto de Maternidad y Ginecología Nuestra Señora de las Mercedes, 4000 San Miguel de Tucumán, Argentina; ^4^Dirección de Investigación, Centro de Educación Médica e Investigaciones Clínicas (CEMIC), 1431 Buenos Aires, Argentina

## Abstract

The amnion is a specialized tissue in contact with the amniotic fluid, which is in a constantly changing state. To investigate the importance of epigenetic events in this tissue in the physiology and pathophysiology of pregnancy, we performed genome-wide DNA methylation profiling of human amnion from term (with and without labor) and preterm deliveries. Using the Illumina Infinium HumanMethylation27 BeadChip, we identified genes exhibiting differential methylation associated with normal labor and preterm birth. Functional analysis of the differentially methylated genes revealed biologically relevant enriched gene sets. Bisulfite sequencing analysis of the promoter region of the oxytocin receptor (*OXTR*) gene detected two CpG dinucleotides showing significant methylation differences among the three groups of samples. Hypermethylation of the CpG island of the solute carrier family 30 member 3 (*SLC30A3*) gene in preterm amnion was confirmed by methylation-specific PCR. This work provides preliminary evidence that DNA methylation changes in the amnion may be at least partially involved in the physiological process of labor and the etiology of preterm birth and suggests that DNA methylation profiles, in combination with other biological data, may provide valuable insight into the mechanisms underlying normal and pathological pregnancies.

## 1. Introduction

 The human amnion is the inner layer of the fetal membranes composed of a monolayer of epithelial cells attached to a basement membrane overlying a collagen-rich stroma [1, 2]. This tissue, which encloses the amniotic fluid, protects the fetus from external mechanical forces and provides an environment that supports fetal movement and growth [3, 4]. The amnion is also a metabolically active tissue involved in the synthesis of various substances with important functions during pregnancy, including prostaglandins and cytokines [1, 5, 6]. It is particularly well known as a major source of prostaglandin E2, a potent molecule mediating cervical ripening and myometrial contraction [7–10], whose levels dramatically increase before and during labor [11, 12].

The amniotic membrane provides most of the tensile strength of the fetal membranes, and alterations in its integrity can lead to undesirable pregnancy outcomes such as preterm premature rupture of membranes (PPROMs) [1, 13], which complicates 3% of all pregnancies and is responsible for approximately one-third of all preterm births (PTBs) [14]. Given the important role of the amnion in the maintenance of pregnancy and parturition, investigation into molecular events occurring in this tissue may contribute to a better understanding of physiological and pathological processes involved in pregnancy.

Considering that the amniotic fluid is in a constantly changing state, it may be critical that the amnion properly responds to environmental cues from the amniotic fluid to accommodate the dynamic needs of the fetus, which could be mediated through epigenetic processes. A previous study by Wang et al. [15] has shown that matrix metalloproteinase 1 (*MMP1*), whose genetic variation is associated with susceptibility to PPROM [16], is regulated at the epigenetic level, specifically by DNA methylation, and that *MMP1* promoter methylation status correlates with its expression in the amnion and association with PPROM. This finding suggests that the amnion represents an intriguing source of tissue for studying epigenetic events of potential physiological and pathological relevance.

In this study, we performed genome-wide methylation profiling of human term and preterm amnion in order to explore the possible importance of DNA methylation in physiologic labor as well as the etiology of PTB. In addition, independent of the genome-wide methylation study, we carried out methylation analysis of the promoter region of the oxytocin receptor (*OXTR*) gene whose role in human parturition is well established [17]. Given that *OXTR* expression in the amnion increases in association with the onset of labor [18] and that its aberrant methylation in other tissue types has been implicated in autism [19], a disorder that has been associated with PTB [20, 21], we sought to investigate if DNA methylation could represent one mechanism regulating *OXTR* gene function in the contexts of normal parturition and prematurity.

## 2. Materials and Methods

### 2.1. Placental Tissue Collection and Preparation

 Fresh human placentas were collected in 2009 and 2010 at the University of Iowa Hospitals and Clinics in IA, USA and Instituto de Maternidad y Ginecología Nuestra Señora de las Mercedes in Tucumán, Argentina with signed informed consent and an institutional review board approval. We examined 121 placentas from three groups of patients undergoing: term cesarean delivery without labor (term no labor (TNL) group, *n* = 18), normal term vaginal delivery (term labor (TL) group, *n* = 40), and spontaneous preterm (<37 weeks of gestation) delivery (preterm labor (PTL) group, *n* = 63). Gestational age (GA) was determined using the first day of the last menstrual period as well as by ultrasound examination and was confirmed by assessment at birth. Each placenta was dissected into fetal (amnion, chorion) and maternal (decidua basalis) components within an hour of delivery. The amnion and chorion obtained from the extraplacental membranes (reflected membranes) were separated by blunt dissection under sterile conditions. Decidual tissue samples were macroscopically isolated from the surface of the basal plate of the placenta. After being cut into small pieces, the dissected tissues were placed in RNA later solution (Applied Biosystems, Carlsbad, CA, USA) and stored per manufacturer's recommendations until used. A subset of these samples was selected for genome-wide methylation analysis on the basis of their informativity in relation to our previous gene expression profiling study (unpublished). Additional samples used for validation experiments were selected primarily based on the quality of DNA or RNA extracted from the tissue samples.

### 2.2. DNA Preparation and Methylation Standards

 Genomic DNA was extracted from placental tissue samples using the DNeasy Blood & Tissue Kit (QIAGEN, Valencia, CA, USA) following the manufacturer's protocol. The quality of the extracted DNA was evaluated by agarose gel electrophoresis. 500 ng of DNA was bisulfite-converted using the EZ DNA Methylation Kit (Zymo Research, Irvine, CA, USA) according to the manufacturer's instructions, and used in subsequent experiments. Universal Methylated Human DNA Standard (Zymo Research), which is enzymatically methylated *in vitro* at all cytosines in CpG dinucleotides, was used as a positive control in the Illumina Infinium methylation assay. We also used Human Methylated and Non-methylated DNA Standards (Zymo Research) as positive and negative controls for methylation-specific PCR. Both of the standards are purified from DNMT1 and DNMT3b double-knockout HCT116 cells, but the methylated standard is enzymatically methylated at all cytosines in CpG dinucleotides.

### 2.3. Genome-Wide DNA Methylation Analysis

#### 2.3.1. Illumina Infinium Methylation Assay

DNA methylation profiling was performed by the W.M. Keck Biotechnology Resource Laboratory at Yale University, using the Illumina Infinium HumanMethylation27 BeadChip (Illumina, San Diego, CA, USA). Details of the design and general properties of this platform have been previously described [22]. A total of 24 samples were assayed on two BeadChips (12 samples per chip) following the standard protocol provided by Illumina. The samples examined included 9 individual and 1 pooled amnion samples each from the TNL and TL groups, one pooled amnion sample from the PTL group obtained by combining 6 individual samples, and 3 controls (methylated DNA control treated with M.SssI methyltransferase (New England Biolabs, Ipswich, MA, USA), Universal Methylated Human DNA Standard (Zymo Research), and bisulfite-untreated control). These samples were selected from among patients who had participated in our previous gene expression profiling study (unpublished), performed independently of the current work. Based on this previous study, which showed heterogeneous global gene expression patterns among PTL samples, we only included one pooled PTL sample to assess a group DNA methylation average. The samples were arranged randomly on each chip and were processed in a blinded fashion.  Table 1 summarizes the clinical characteristics of the three groups of samples studied.

#### 2.3.2. Quality Control and Statistical Analysis

Data analysis was conducted on a fee-for-service basis by the W.M. Keck Biostatistics Resource at Yale University with GenomeStudio Methylation Module v1.0 (Illumina). We evaluated the quality of the data based on the signals of assay built-in control probes (staining, hybridization, target removal, extension, bisulfite conversion, methylation signal specificity, background determination, and overall assay performance) and three experimental controls (two positive methylated controls and one non-bisulfite-converted control), and confirmed the reliability of our data. Principal component analysis (PCA) demonstrated that there is no significant batch effect among the three groups of samples examined. The methylation status of each interrogated CpG site was determined employing the *β*-value (defined as the fraction of methylation, calculation details described in a previous study [23]) method. An average *β*-value (AVG_Beta) for each CpG locus ranging from 0 (unmethylated) to 1 (completely methylated) was extracted utilizing the GenomeStudio software and used in further analyses. For the determination of differential methylation between two given groups, we used the Illumina custom error model. This model assumes a normal distribution of the methylation value (*β*) among replicates corresponding to a set of biological conditions (TNL, TL, and PTL). We prioritized differentially methylated CpG sites by difference score (DiffScore). DiffScore, which takes into account background noise and sample variability [24], was calculated using the following formula: DiffScore = 10sgn⁡(*β*_condition_ − *β*_reference_)log⁡_10_ *P*, where *β*_condition_ = *β*_TL_/*β*_PTL_, *β*_reference_ = *β*_TNL_ or *β*_condition_ = *β*_TNL_/*β*_PTL_, *β*_reference_ = *β*_TL_. The resulting differentially methylated CpG sites were annotated with respect to their nearest gene based on the information provided by Illumina. A more detailed description of the Illumina custom error model and the DiffScore has been provided previously [25].

#### 2.3.3. Functional Enrichment Analysis

Differentially methylated genes (DMGs) with a DiffScore of >20 (equivalent to *P*-value of <0.01) were evaluated for functional enrichment using predefined gene sets from the Molecular Signatures Database (MSigDB) [26]. We searched for significantly enriched gene sets by computing overlaps between the lists of DMGs and the CP collection (canonical pathways, 880 gene sets) or the C5 collection (GO gene sets, 1454 gene sets) in the MSigDB. Gene sets with a *P*-value (based on the hypergeometric distribution) less than 0.05 were considered significant.

### 2.4. Bisulfite Sequencing (BS)

To validate methylation differences revealed by the genome-wide methylation assay, we performed bisulfite sequencing on urocortin (*UCN*), a gene identified as differentially methylated between the TL and PTL groups, and *OXTR*, a gene whose methylation status has recently been shown to be important in the pathogenesis of autism [19]. We investigated the methylation status of *OXTR*, given its significant role in parturition [17] and its labor-associated expression pattern in the amnion [18], which makes it a potential candidate gene for PTB. There are two *OXTR* CpG sites targeted by the Illumina Infinium BeadChip assay, both of which were not identified as being differentially methylated. However, because there is currently no evidence supporting the biological importance of the regions containing the two sites, we focused our BS analysis on CpG sites of known biological significance that are located in a different region of the *OXTR* gene. Primers for *UCN* were designed to cover the CpG site identified as being differentially methylated by genome-wide methylation profiling, using the default parameters of MethPrimer [27]. PCR amplification using the primer pair results in a 278 bp product that spans part of the promoter, exon 1, and part of intron 1 of *UCN* (-439 to -162 relative to translation start site (TSS)) containing 16 CpG sites. For *OXTR*, we used the same primers and PCR conditions as those used in the previous study [19]. PCR amplification using the primer set results in a 358 bp product that spans the *OXTR* promoter (-1195 to -838 relative to TSS) containing 22 CpG sites that has been associated with tissue-specific *OXTR* expression [28] and the development of autism [19]. The regions examined in both genes were located within CpG islands. We carried out our analysis using the same samples assayed on the BeadChips and eight additional independent PTL samples (TNL, *n* = 9; TL, *n* = 9; PTL, *n* = 14). Bisulfite-converted DNA was PCR-amplified using ZymoTaq DNA polymerase (Zymo Research). The resulting PCR products were run on an agarose gel and cloned into the pGEM-T Easy vector (Promega, Madison, WI, USA). Individual clones were isolated, amplified following standard protocols, and purified using the PureLink Quick Plasmid Miniprep Kit (Invitrogen, Carlsbad, CA, USA) per manufacturer's instructions. Ten clones per sample, on average, were isolated and sequenced at the University of Iowa DNA facility. Percentage methylation was determined for each CpG site similarly as done in previous work [19]. Statistical analysis was conducted using SigmaPlot 11.0 (Systat Software, San Jose, CA, USA). Significance of differential methylation (DM) was assessed using the  *t*-test (two-tailed), Mann-Whitney (M-W) rank sum test (two-sided), one-way ANOVA, or Kruskal-Wallis (K-W) one-way ANOVA by ranks, as indicated in the text and/or figure legends. Post hoc analysis following ANOVA was performed using either the Holm-Sidak or Dunn's Method. A *P* < 0.05 was considered significant.

### 2.5. Encyclopedia of DNA Elements (ENCODE) ChIP-Seq Data

We examined the potential functional significance of the region of the *OXTR* gene containing CpG sites with statistically different DNA methylation status (CpGs-959 and -1084) using the ChIP-seq data from the ENCODE project available in the University of California Santa Cruz (UCSC) genome browser [29, 30]. We specifically used the suppressor of zeste 12 homolog (Drosophila) (*SUZ12*) and *Pol2* ChIP-seq data generated by the laboratories of Michael Snyder at Stanford University and Vishy Iyer at the University of Texas Austin. The ChIP-Seq data were obtained using human cells (NT2-D1 for the *SUZ12* data; GM18526, 18951, 19099, 19193, and ProgFib for the *Pol2* data).

### 2.6. Methylation-Specific PCR (MSP)

Validation of DM was additionally carried out using methylation-specific PCR (MSP). Two pairs of primers (unmethylated and methylated) for each of the lysophosphatidic acid receptor 5 (*LPAR5*), paternally expressed 10 (*PEG10*), and solute carrier family 30 member 3 (*SLC30A3*) genes were designed using the MSP-specific default parameters of the MethPrimer program [27]. Bisulfite-converted DNA extracted from amnion tissues (TNL, *n* = 9; TL, *n* = 9; PTL, *n* = 14) was PCR-amplified using Biolase DNA polymerase (Bioline, Taunton, MA, USA). The resulting PCR products were visualized on a 2% agarose gel. Human Methylated and Non-methylated DNA Standards from Zymo Research were used as positive and negative controls.

### 2.7. RNA Extraction and Real-Time qRT-PCR

Total RNA was extracted from amnion (TNL, *n* = 14; TL, *n* = 34; PTL, *n* = 59) and decidua (TNL, *n* = 12; TL, *n* = 16; PTL, *n* = 31) tissues using TRIzol reagent (Invitrogen) according to the manufacturer's protocol. The quality of extracted RNA was checked using the Agilent 2100 Bioanalyzer (Agilent Technologies, Santa Clara, CA, USA). Reverse transcription was carried out with the High Capacity cDNA Reverse Transcription Kit (Applied Biosystems), using random hexamers as primers following the manufacturer's instructions. Real-time qRT-PCR was performed using synthesized cDNA as a template, gene-specific primers (*UCN* and *OXTR*) and Power SYBR Green PCR Master Mix (Applied Biosystems). The reactions (including no-template controls) were run in triplicate on the 7900HT Fast Real-Time PCR System (Applied Biosystems) using *ACTB* (beta actin) [31] as an endogenous reference. Data were analyzed with the SDS 2.4 software (Applied Biosystems), employing the comparative CT method [32]. Absence of nonspecific amplification was confirmed by dissociation curve analysis. Samples with a value that falls outside ±2 standard deviations of the group mean were defined as outliers and removed from the study. Statistical analysis was performed similarly as described above in the bisulfite sequencing section. Data were presented as mean ± standard error of the mean (SEM).

## 3. Results

### 3.1. Genome-Wide Patterns of DNA Methylation and Differentially Methylated CpG Loci between Term (Non-Labored and Labored) and Preterm Amnion Tissues

**Figure 1:**
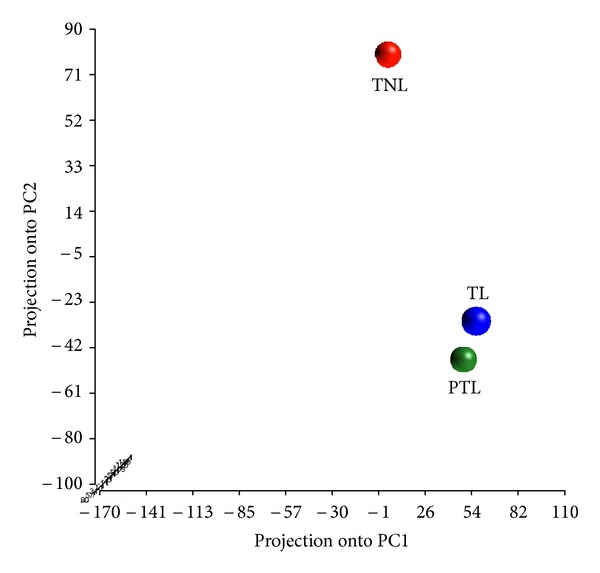
Principal component analysis (PCA) plot of DNA methylation profiles in term (non-labored and labored) and preterm amnion. Each colored dot represents a pooled DNA sample from term no labor (TNL), term labor (TL), or preterm labor (PTL) group. Note that the TNL sample is placed distantly from the TL or PTL samples, indicating that the TNL group displays distinctly different methylation patterns compared to the other two groups.

To investigate the possible involvement of epigenetic mechanisms in the physiology of normal labor and the pathogenesis of PTB, we examined the genome-wide methylation profiles of the amnion obtained following term (TNL and TL, *n* = 9 for each) and preterm (PTL, *n* = 6) deliveries using the Illumina Infinium BeadChip platform. The overall levels of DNA methylation in the experimental samples were low with third quartile AVG_Beta values between 0.4 and 0.55. Principal component analysis (PCA) placed the pooled TL and PTL samples close to each other and very distant from the pooled TNL sample (Figure 1), which indicates that the genome-wide methylation patterns in amnion tissues from the two spontaneous labor groups (regardless of gestational age (GA) at delivery) are more similar to each other than to those observed in non-labor tissues.

We also performed gene/locus level analysis of differential methylation (DM), searching for methylation changes associated with labor and/or PTB at specific CpG sites. Using the Illumina custom error model algorithm, we identified 65 CpG sites in 64 and 61 autosomal genes each that are differentially methylated between the TNL and TL groups and the TL and PTL groups, respectively with a DiffScore of >30 (equivalent to *P*-value of <0.001). Listed in Table 2 are the 15 most highly differentially methylated genes (DMGs). It was noted that among the genes with differentially methylated sites, although very few, were those belonging to special classes of genes, including noncoding RNAs and imprinted genes (such as Down syndrome critical region gene 10 (*DSCR10*), FBXL19 antisense RNA 1 (*FBXL19-AS1*), and paternally expressed 10 (*PEG10*) as shown in Table 2), many of which have regulatory functions in diverse biological processes.

### 3.2. Functional Enrichment Analysis

To determine the biological significance of DMGs, functional annotation analysis was performed. Our approach involved examining the extent of overlap between our lists of DMGs and predefined annotated gene sets from the MSigDB [26] (see Section 2 for further details). For this analysis, we used gene lists with a less stringent *P*-value cutoff of <0.01 (corresponding to a DiffScore of >20), given the small number of DMGs (*n* = 65) with a *P*-value below 0.001. We found that 7 gene sets were significantly overrepresented (*P* < 0.05) in the list of 110 DMGs between the TNL and TL groups. The seven enriched gene sets included cation transport, ion channel activity, and those shown in Table 3, most of which are highly relevant to molecular processes involved in physiologic labor. Among the 186 DMGs between the TL and PTL groups, 17 gene sets were overrepresented. Many of the enriched gene sets were found to be associated with the regulation of cell behavior and extracellular matrix-cell interactions, including focal adhesion, cell junction, cell-substrate adherens junction, and integrin binding (Table 3).

### 3.3. Bisulfite Sequencing (BS) Analysis of Differential Methylation

**Figure 2:**
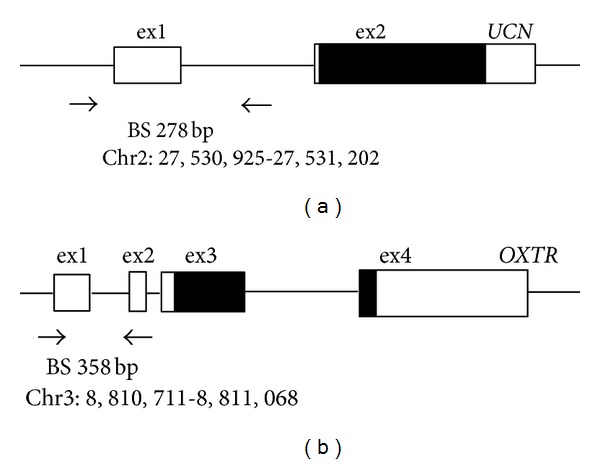
Schematic representation of CpG island regions of *UCN* (a) and *OXTR* (b) analyzed by bisulfite sequencing (BS). Black horizontal arrows denote BS PCR primer binding sites. Solid box: coding region; open box: untranslated region. The expected PCR product sizes and positions of the primer binding sites (chromosome and base count, NCBI Build GRCh37/hg19) are indicated. Further details on the PCR-amplified regions are provided in Section 2.

 To validate DM detected by genome-wide methylation profiling, we performed BS analysis on *UCN*, a gene identified as being overmethylated in the PTL group compared with the TL group with a DiffScore >50 (Table 2). We performed the same analysis on one additional gene named oxytocin receptor (*OXTR*) whose mRNA and protein expression has been shown to be markedly upregulated in association with labor in primary human amnion epithelial cells [18]. Previous studies have demonstrated that the methylation status of the promoter region of this gene is associated with tissue-specific *OXTR* expression [28] and the development of autism [19], a disorder linked to PTB [20, 21, 33, 34]. These findings intrigued us to investigate whether DNA methylation could represent one mechanism regulating the labor-associated activity of *OXTR* in the amnion. We selected the two genes (*UCN* and *OXTR*), given their crucial role in normal labor and parturition, which makes them potential candidate genes for PTB. Details on the regions amplified, samples used in the BS experiments, and statistical tests performed for the analysis of the sequencing results are given in Section 2 and Figure 2.

All 16 CpG dinucleotides interrogated in the *UCN* gene showed some degree of methylation with the ones at positions -361, -335, and -319, being more highly methylated (22.9–55.7%, Table 4) compared with those at other positions (1.1–17.1%). All except two CpG sites were overmethylated in the PTL samples compared to the TL samples, showing the expected direction of DM. However, the differences were not statistically significant.

 For *OXTR*, since we had no *priori* data on the methylation status of the 22 CpG sites in amnion tissue, all three groups of samples (TNL, TL, and PTL) were examined. Consistent with the finding of Gregory et al. [19], 5 CpG sites at positions -959, -934, -924, -901, and -860 showed the highest levels (22.2–68.6%, Table 4) and variation in methylation, whereas very little or no methylation (0–5.7%) was observed at the other sites. We found that one (CpG-959) of the five sites was significantly differentially methylated among the three groups tested (one-way ANOVA, *P* = 0.014, Table 4). Pairwise comparisons (Holm-Sidak test) revealed significant differences between the TNL and TL groups (*P* = 0.017) and the TNL and PTL groups (*P* = 0.025) and borderline significant difference between the TL and PTL groups (*P* = 0.050), demonstrating more distinct differences in methylation at this site between non-labor and labor tissues than between term and preterm tissues.

 To determine if the observed DM also occurs in other parts of the placenta where the genes are known to be expressed [17, 35], we extended our study to decidua tissues from the same groups of individuals. The decidua, which is of maternal origin, unlike the amnion of fetal origin [4], was selected, given that the function of *OXTR* in parturition has been well demonstrated in maternal tissue [17], and therefore, the examination of the decidua, along with the amnion, may allow us to compare the methylation state of the *OXTR* gene and possibly its importance in both fetal and maternal tissues.

The overall methylation patterns observed in the decidua were similar to those identified in the amnion. However, unlike in the amnion tissues, the methylation levels not at CpG-959, but at different sites (CpGs-934 and -1084), were found to be statistically significantly different (*P* = 0.02, 0.008, resp., K-W one-way ANOVA by ranks) among the three groups of the decidua tissues (Table 4). The CpG-1084 site, interestingly, was completely unmethylated in the TL group, whereas it was methylated to some small degree in the other two groups (TNL, 10%; PTL, 8.7%) (Table 4). Significant differences between the TL and TNL or PTL groups were confirmed by Dunn's post hoc test (*P* < 0.05). In the case of CpG-934, the difference was significant only between the TL and PTL groups. Taken together, it appears that there exist compartment-specific *OXTR* methylation patterns in the placenta.

### 3.4. Analysis of *UCN* and *OXTR* Gene Expression in the Amnion and Decidua

**Figure 3:**
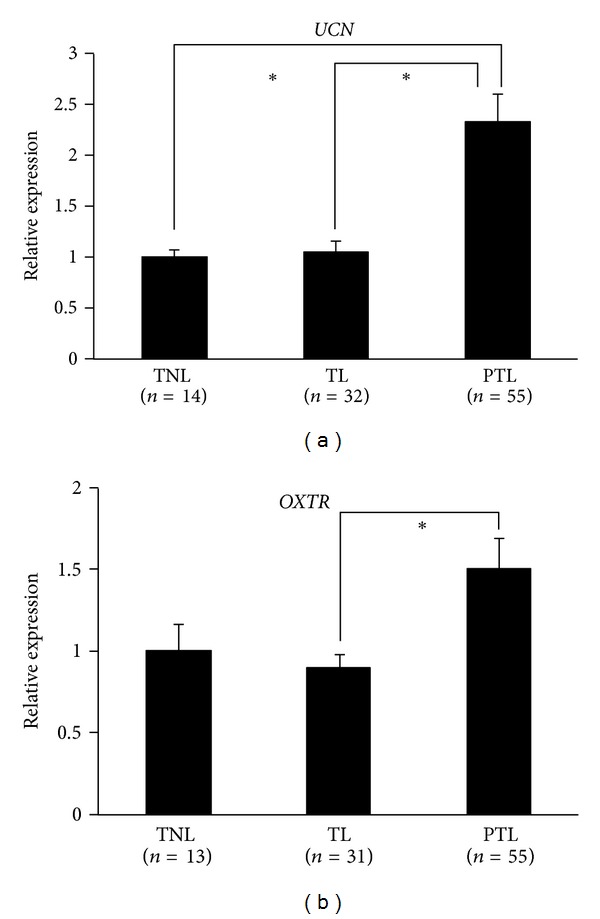
*UCN *and* OXTR *mRNA expression levels in term (non-labored and labored) and preterm amnion. Expression levels were normalized to that of beta-actin* (ACTB). *Experiments were performed in triplicate. Data presented are mean ± standard error of the mean (SEM). Asterisks represent statistically significant differences (*P* < 0.05, K-W one-way ANOVA by ranks followed by Dunn's post hoc test) between specified groups.

 To evaluate the functional significance of the methylation status of the two genes, we performed gene expression analysis using qRT-PCR on an extended set of amnion and decidua tissues (*n* = 107, 59, resp.) from the three groups. Although the DM of *UCN* was not validated by BS, we observed a statistically significant 2.3-fold increase in its transcript levels in the PTL amnion samples compared to the TNL and TL samples (*P* < 0.001, K-W one-way ANOVA by ranks, Figure 3). There was also a statistically significant, but less than twofold increase in *OXTR* mRNA levels in the PTL amnion samples compared with the TL samples (*P* < 0.05, K-W one-way ANOVA by ranks, Dunn's post hoc test, Figure 3). The results were not replicated in the decidua samples for either gene. These findings suggest that the upregulation of *UCN* is specific to the amnion from spontaneous preterm deliveries, and that the DM observed in *OXTR* may not correlate with *OXTR* expression given that methylation generally plays a role in gene silencing.

### 3.5. Methylation-Specific PCR (MSP) Analysis of Differential Methylation

**Figure 4:**
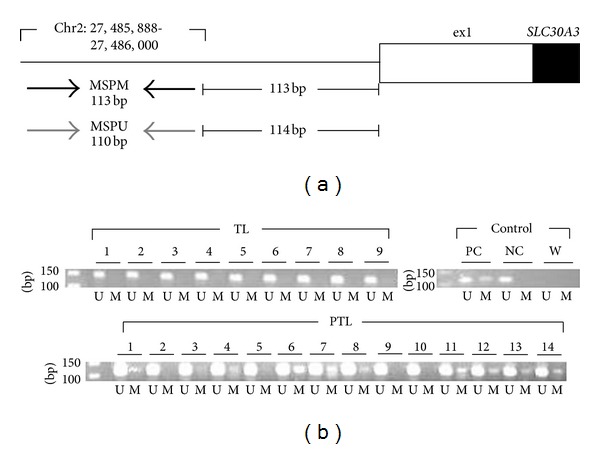
Methylation-specific PCR (MSP) analysis of *SLC30A3*. (a) Schematic representation of MSP primer binding sites. Black horizontal arrows: methylated-specific primer (MSPM) binding sites; gray horizontal arrows: unmethylated-specific primer (MSPU) binding sites. The expected PCR product sizes and positions of the primer annealing sites (chromosome and base count, NCBI Build GRCh37/hg19) are indicated. Solid box: coding region; open box: untranslated region. (b) Agarose gel electrophoresis of MSP products. M: product amplified with MSPM; U: product amplified with MSPU. PC: positive control, human methylated DNA standard; NC: negative control, human unmethylated DNA standard; W: water control (details can be found in Section 2). Note that the TL samples show only unmethylated PCR products, while many of the PTL samples show both methylated and unmethylated PCR products, indicative of partial methylation.

As an alternative approach to validate DNA methylation differences captured by our genome-wide methylation study, we carried out methylation-specific PCR (MSP) for selected 3 DMGs between the TNL and TL groups (*PEG10*) and between the TL and PTL groups (*LPAR5* and *SLC30A3*). Analysis of the same set of amnion samples used in BS revealed no intergroup differences in *PEG10* and *LPAR5* methylation (data not shown). However, the methylation status of *SLC30A3* was in good agreement with our genome-wide methylation data with methylated MSP products present in 10 out of 14 (71%) PTL samples and none of the TL samples (Figure 4).

## 4. Discussion

The present study investigated if there exist unique genome-wide methylation signatures that distinguish among term (non-labored and labored) and preterm amnion tissues. Our methylation profiling revealed a higher degree of similarity between the methylation patterns in the TL and PTL pooled samples than those observed in the TNL pooled sample, suggesting the potential role of methylation in the regulation of labor, independent from GA. We identified a relatively small number of DMGs between the TNL and TL groups and the TL and PTL groups (65 genes each) at the *P* < 0.001 significance level. This observation may be attributed to the small sample size and the sample-to-sample variability related to GA. Gene set enrichment analysis of those genes revealed significant overrepresentation of pathways that appear to be functionally relevant (Table 3). The enrichment of pathways related to ion transport, ion channel activity, and cytokine production among the DMGs between the TNL and TL groups reflects biochemical and molecular events associated with the onset of labor, which, along with hormonal factors, help to initiate parturition. These results are at least partially in line with previous gene expression profiling studies reporting labor-associated cytokine-related gene signatures in human amniotic [36] and chorioamniotic [37] membranes. The overrepresentation of heart-(development and contraction) related gene sets may be explained by the presence of myofibroblasts in the connective tissue of the amnion [38], which have contractile ability [39], and hence are involved in heart rhythm regulation [40] and, possibly, prevention of excessive distension of the amniotic membrane [38]. The DMGs between the TL and PTL groups were enriched in gene sets involved in cell adhesion, cell-cell and cell-extracellular matrix interactions, which have crucial roles in the modulation of cellular behavior and tissue maintenance and organization [41]. This observation confirms the importance of intact fetal membranes as a critical factor in the maintenance of pregnancy. Another overrepresented gene set was the negative regulation of transferase activity. Given the versatile roles of transferases, differential methylation of this group of genes (including *HEXIM1*, *SFN*, *CBLC*, and *DUSP2*) may influence a wide range of cellular processes in a way that interferes with timely onset of labor and parturition. Among these genes, *DUSP2* has previously been documented as being significantly upregulated following interleukin-1*β* (IL-1*β*) stimulation in myometrial cells [42], suggesting its potential role in the mediation of uterine contractions. It would be intriguing to examine how the activity of *DUSP2* in the amnion may contribute to the process of parturition.

Our study at the individual gene level using BS revealed three CpG sites (CpGs-934, -959, and -1084) in *OXTR* that exhibit significant DM among the three groups of amnion and decidua tissues, which are of fetal and maternal origin, respectively [4]. Subsequent gene expression analysis demonstrated no correlation between gene expression and methylation and therefore, the functional significance of the observed DM remains undetermined. Previous work showed that site-specific methylation can result in transcriptional alterations through its effects on the interaction of transcription factors (TFs) with its cognate DNA sequence [43]. Currently, there are no known TF binding sites around CpG-959, which was previously identified as significantly hypermethylated in peripheral blood mononuclear cells from autistic patients compared with those from control patients [19]. However, Gregory et al. [19] have indicated that CpG-934, whose differential methylation has also been associated with autism, falls within predicted binding domains for v-rel reticuloendotheliosis viral oncogene homolog (avian) (c-Rel), zinc fingers and homeoboxes 2 (ZHX2), and lectin, galactoside-binding, soluble, 4 (LGALS4). Using ENCODE ChIP-seq data available in the UCSC genome browser, we also found that CpG-1084 falls within putative binding sites for *SUZ12* and *Pol2* (see Section 2 for more details), which warrants future studies to dissect the impact of the methylation status at this specific dinucleotide on the interactions between these TFs and their binding sites.

Despite the lack of any significant difference in *UCN* methylation levels between the TL and PTL groups, our observation of a significant, more than 2-fold increase in *UCN* mRNA levels in the PTL amnion tissues compared with the term tissues suggests a potential role of this gene in the etiology of PTB, which encodes an endogenous ligand for corticotropin releasing hormone receptor (CRHR) that mediates the action of CRH, one of the major endocrine factors in parturition [44]. Given that there are several putative binding sites for TFs (such as C7EBP, GATA, and MyoD) [45] upstream of the region examined in this study, it would be intriguing to investigate whether the methylation status of CpG dinucleotides encompassing those sites correlates with the observed gene expression patterns. It would also be worthwhile to examine if mechanisms other than methylation underlie the transcriptional regulation of *UCN* in the amnion.

Our MSP analysis identified another gene (*SLC30A3*) that might play a role in pathogenic processes of PTB. This gene, also known as *ZNT3*, encodes a zinc transporter responsible for zinc efflux from the cytoplasm to extracellular spaces or intracellular organelles [46]. Given the differential expression of *SLC30A3* in relation to dietary zinc and/or glucose supply in mouse placenta [47] and beta cells [48], it is postulated that its dysregulated expression due to aberrant methylation in human amnion may influence nutritional homeostasis during pregnancy, ultimately, leading to PTB.

Our work was limited by the small sample size and the lack of control for gender-specific methylation differences [49, 50]. Another major limitation is that the PTL tissues were examined as a pooled sample, not individually. Previous studies have demonstrated that pooled DNA samples can be used to provide a reliable estimate of average group methylation when analyzed using high-throughput techniques such as MALDI-TOF mass spectrometry [51, 52]. Therefore, a DNA pooling approach using such systems could be employed in future studies for large-scale assessment of methylation variations in maternal and fetal tissues. Very recently, it has been shown that neonatal DNA exhibits a considerable degree of GA-associated variability in DNA methylation patterns [53]. Given this finding, a precisely stratified analysis based on GA may allow a more accurate characterization of DNA methylation profiles associated with term and preterm pregnancies.

## 5. Conclusion

This work provides preliminary evidence that DNA methylation changes may play at least a partial role in physiologic labor and the etiology of PTB, and suggests that DNA methylation profiles, together with other types of biological data, hold a promise for the identification of genes involved in normal parturition and preterm birth.

## Figures and Tables

**Table 1:** Clinical characteristics of the three subject groups studied by genome-wide DNA methylation profiling.

Parameter	TNL (*n* = 9)^1^	TL (*n* = 9)^1^	PTL (*n* = 6)^2^
Gestational age (weeks)^3^	39.1 ± 0.8	38.8 ± 0.8	33.5 ± 2.6
Race			
White	4	3	3
Black	0	0	1
Other	5	6	2
Maternal age at delivery (years)^3^	29.7 ± 5.5	27 ± 4.4	28.7 ± 3.5
	(Range 22–38)	(Range 20–33)	(Range 25–33)
Antibiotics during pregnancy or labor			
Yes	6	1	5
No	3	7	0
Unknown	0	1	1
Birth weight (grams)^3^	3508.3 ± 267.2	3354.1 ± 348.6	2102.2 ± 724.6
Infant gender			
Female	6	4	2
Male	3	5	4

^1^Examined both individually and as a pooled sample.

^2^Examined as a pooled sample.

^3^Data are presented as mean ± standard deviation (SD).

Abbreviations: TNL: term no labor; TL: term labor; PTL: preterm labor.

**Table 2:** List of top 15 differentially methylated autosomal genes in amnion tissues from term (TNL, TL) and preterm (PTL) deliveries ranked by statistical significance^1^.

TNL versus TL^2^			TL versus PTL^3^		
Gene	Locus	CpG island^4^	Gene	Locus	CpG island^4^
*IL32 *	16p13.3	No	*TOB1 *	17q21	No
*EDARADD *	1q42.3	No	*PNPLA3 *	22q13.31	No
*STK19 *	6p21.3	No	*ZNF671 *	19q13.43	No
*EXTL1 *	1p36.1	No	*DAB2IP * ^ 7^	9q33.1–q33.3	No
*HLA-DQB2 *	6p21	No	*MFNG *	22q12	No
*MFSD3 *	8q24.3	Yes	*UCN *	2p23–p21	Yes
*RAB31 *	18p11.3	Yes	*EXOC3L2 *	19q13.32	Yes
*PNPLA3 *	22q13.31	No	*SLC44A2 *	19p13.1	Yes
*GRHPR *	9q12	Yes	*FBXL19-AS1 * ^ 6^	16p11.2	Yes
*MPHOSPH10 *	2p13.3	No	*DLGAP5 *	14q22.3	Yes
*PEG10 * ^ 5^	7q21	No	*SLC30A3 *	2p23.3	Yes
*DSCR10 * ^ 6^	21q22.13	No	*CHFR *	12q24.33	No
*SRRD *	22q12.1	Yes	*C11orf1 *	11q23.1	No
*POLI *	18q21.1	Yes	*SLC24A4 *	14q32.12	No
*OSTalpha *	3q29	No	*PI4KB *	1q21	No

^1^Statistical significance was determined based on *P*-values calculated from DiffScores. All genes listed here have a DiffScore >40 (corresponding to *P*-value of <0.0001).

^2^Genes most highly methylated in the TL group compared to the TNL group.

^3^Genes most highly methylated in the PTL group compared to the TL group.

^4^Defined by the CpG island track in the UCSC Genome Browser.

^5^An imprinted gene.

^6^Non-protein coding genes.

^7^A gene identified as having three non-island CpG sites with a DiffScore >40.

Abbreviations: TNL: term no labor; TL: term labor; PTL: preterm labor.

**Table 3:** Gene sets overrepresented among differentially methylated genes in amnion tissues from term (TNL, TL) and preterm (PTL) deliveries^1^.

TNL versus TL	
Gene set^2^	*P*-value^3^
HEART_DEVELOPMENT	0.012
POSITIVE_REGULATION_OF_CYTOKINE_PRODUCTION	0.017
GATED_CHANNEL_ACTIVITY	0.024
REGULATION_OF_HEART_CONTRACTION	0.041
REGULATION_OF_CYTOKINE_PRODUCTION	0.044

TL versus PTL	

NEGATIVE_REGULATION_OF_TRANSFERASE_ACTIVITY	0.007
ADHERENS_JUNCTION^4^	0.011
HEPARIN_BINDING	0.014
FOCAL_ADHESION_FORMATION	0.02
FOCAL_ADHESION	0.024

^1^Presented are the top 5 most significantly enriched gene sets from C5 collection (GO gene sets).

^2^Defined in the Molecular Signatures Database (MSigDB).

^3^The cutoff for statistical significance was *P* = 0.05.

^4^Also identified as being enriched (*P* = 0.049) in the analysis performed with the CP collection.

Abbreviations: TNL: term no labor; TL: term labor; PTL: preterm labor.

**Table 4:** *UCN* and *OXTR* promoter methylation status in the amnion and decidua from term (TNL, TL) and preterm (PTL) deliveries^1^.

*UCN *								
Site^2^	Amnion				Decidua			
	TL		PTL	*P*-value	TL		PTL	*P*-value
-190^3^	2.2%		8.6%	0.39	6.7%		14.8%	0.07
-279^3^	2.2%		11.4%	0.07	15.6%		12.5%	0.97
-319^4^	24.4%		22.9%	0.83	19.9%		30.8%	0.08
-335^4^	23.3%		27.1%	0.56	23.2%		25.8%	0.75
-361^4^	48.9%		55.7%	0.33	32.1%		36.4%	0.48

*OXTR *								
Site^2^	Amnion				Decidua			
	TNL	TL	PTL	*P*-value	TNL	TL	PTL	*P*-value

-860	24.4%	22.2%	24.3%	0.96	20%	15.6%	24.6%	0.19
-901	37.8%	30%	45.7%	0.17	45.6%	38.9%	46.9%	0.66
-924	56.7%	62.2%	68.6%	0.29	60%	52.2%	67.2%	0.09
-934^5^	50%	41.1%	59.3%	0.22	46.7%	42.2%	57.1%	0.02
-959^5^	43.3%	24.4%	27.1%	0.014	33.3%	33.3%	30.6%	0.91
-1084^5^	4.4%	4.4%	5.7%	0.97	10%	0%	8.7%	0.008

^1^Presents average % methylation at each CpG site.

^2^Nucleotide positions relative to translation start site.

^3^CpG sites in *UCN* with the lowest *P*-value in each tissue type.

^4^CpG sites methylated at higher levels in both tissues than the average methylation level of all sites examined.

^5^CpG sites with statistically significant (*P* < 0.05) differential methylation in either tissue. Details of statistical tests used are described in Section 2.

Abbreviations: TNL: term no labor; TL: term labor; PTL: preterm labor.
